# Why should we care about social media codes of conduct in healthcare organisations? A systematic literature review

**DOI:** 10.1007/s10389-023-01894-5

**Published:** 2023-04-10

**Authors:** Gitte Galea, Ritesh Chugh, Jo Luck

**Affiliations:** 1 School of Engineering and Technology, Central Queensland University, 45 Abbott Street, Cairns, QLD 4879 Australia; 2 School of Engineering and Technology, Central Queensland University, Bruce Highway, North, Rockhampton, QLD 4702 Australia

**Keywords:** Social media, Code of conduct, Health, Healthcare employee, Healthcare organisations

## Abstract

**Background:**

The conduct of healthcare organisation employees on social media can impact both their personal reputation and that of the organisation. However, social media has blurred the lines between professional and personal communication, and what is acceptable and ethical conduct is not always clear. Furthermore, the global COVID-19 pandemic has changed how healthcare organisations and their employees approach the use of social media, expediting the need to ensure that employees communicating health-related information adhere to employee codes of conduct.

**Aims:**

This review aims to investigate the challenges associated with healthcare organisation employees’ use of social media for sharing health-related information, identify the crucial elements for inclusion in social media codes of conduct for healthcare organisations, and examine the enablers for good codes of conduct.

**Methods:**

A systematic review of the literature from six research database platforms on articles related to codes of conduct addressing the use of social media for healthcare organisation employees was conducted. The screening process yielded 52 articles.

**Results:**

The key finding in this review focuses on privacy, protecting both patients and healthcare organisation employees. While maintaining separate professional and personal social media accounts is a much-discussed approach, training and education on social media codes of conduct can clarify acceptable behaviour both personally and professionally.

**Conclusion:**

The results raise essential questions about healthcare organisation employees’ use of social media. It is evident that organisational support and a constructive culture will enable healthcare organisations to fully realise the benefits of using social media.

## Introduction

Social media has become an integral part of everyday life for people worldwide, and the use of social media in the healthcare landscape is here to stay (Kotsenas et al. 2018a, b). Furthermore, COVID-19 has forced government authorities to quickly and effectively communicate healthcare information to the public, which has expedited the need for healthcare organisations to use social media to disseminate information rapidly. Not limited to the COVID-19 pandemic, healthcare organisations provide a wide variety of health-related community information on social media to engage with and educate the public, such as health promotion and health education, clinic availability, experiential storytelling, employee recruitment and health facts. However, for health communication to be effective, the public must be willing to listen to and act on information received and accept that information from healthcare organisations on social media is truthful and honest. Inappropriate use of social media is a behaviour that may see healthcare organisations’ employees penalised, where the employee is identifiable, and this could damage the healthcare organisations public image (Health and Care Professionals Council 2020). Building trust through consistent ethical conduct on social media will add value to health communication, and codes of conduct have a critical role to play.

Codes of conduct have existed in the healthcare landscape for some time now, with healthcare organisation employees being educated in ethical conduct via global health training programs (Crump and Sugarman 2010). Protecting patient privacy and confidentiality is essential (Panahi et al. 2016). Healthcare organisation employees are perceived as being focused on the health of others and are generally trusted members of the community. However, Collings-Hughes et al. (2021) concluded that most healthcare organisation employees do not think they know the content of the codes of conduct, despite being in a field that values codes, creating a gap where research is needed to create well-written and better-communicated codes.

At the time of writing, a simple Google search such as ‘nurse fired over social media’ revealed over 100 news articles about nurses being terminated for inappropriate posts on social media. This phenomenon is not new. For over a decade, inappropriate posts on social media have resulted in healthcare organisation employees being terminated. In a study by Clark and Kearns (2010), personal discussions on social media concerning hospital patients were seen as a violation of privacy policies.

The adoption and use of social media in healthcare, as part of a business strategy, lags behind many other industries (Kotsenas et al. 2018b). Healthcare organisations face challenges adapting to new information distribution channels as the power of social media grows (Zelmer 2012). Current codes of conduct may not extend to new problems in the use of social media, and some consider social media a threat to employee conduct (Cowin et al. 2019). Healthcare organisations are public entities and are constantly scrutinised, and misinformation posted on social media can affect health decisions (Peek et al. 2015). The blur between personal and professional communication in healthcare can create compromising situations (Peluchette et al. 2016). While a code of conduct on social media is intended to protect a healthcare organisation’s reputation, privacy and productivity, it should not interfere with employees’ personal rights (Popper-Giveon et al. 2019). Hence, an open and flexible approach to the use of social media is required, with a particular focus on embracing changing conditions (Chugh and Joshi 2020).

In the early days of social media, guidelines for how healthcare organisation employees should conduct themselves on social media have been researched and discussed (Ly and Ratnaike 2011; Maloney et al. 2014; Moses et al. 2014; Osis and Pelling 2015), and guidelines and policies have been published (Hughes 2012; Peate 2015). The use of social media in healthcare is still in its infancy or lessons learned phase, and improvements to codes of conduct and policies are needed. There is a research gap of insufficient good policies and practices in the use of social media and what behaviours on social media should be adopted by healthcare organisation employees (Corniati et al. 2019). Hence, this literature review investigates important questions about the use of social media by healthcare organisation employees by focusing on the problems encountered, what should be included in a social media code of conduct and how to enable good social media use. This review concentrates on healthcare organisation employees such as doctors, nurses, physiotherapists, paramedics, and operational and administration employees. It is not limited to a particular type of employee. Put simply, the focus is on employees who work for healthcare organisations and how they conduct themselves on social media, whether for personal or employee use. By reviewing existing literature, the aim is to understand the challenges of using social media for healthcare organisation employees, how codes of conduct can address issues so that social media can be used effectively and how conduct on social media may change for healthcare organisation employees in the future. The specific research questions guiding this literature review are:

- RQ 1. What problems can occur when healthcare organisation employees post health-related information either personally or professionally?
- RQ 2. What elements should be included in healthcare organisations’ social media codes of conduct?
- RQ 3. What are the enablers for the effective use of codes of conduct for healthcare organisations?

The findings and discussion in this review are presented after the research method in three subsections aligned with the research questions. Firstly, the problems are examined, followed by the elements of social media code and the enablers for good codes of conduct.

## Research method

Systematic literature reviews take a clearly formulated approach that can be replicated, and this methodical approach increases the credibility and trustworthiness of the results (Turnbull et al. 2023; Rother 2007). Systematic literature reviews have become increasingly important in the healthcare landscape, as they are often used as a starting point for developing guidelines (Moher et al. 2009). By reviewing previous research, knowledge can be gained, lessons learned, and evidence-based decisions can be made.

**Fig. 1:**
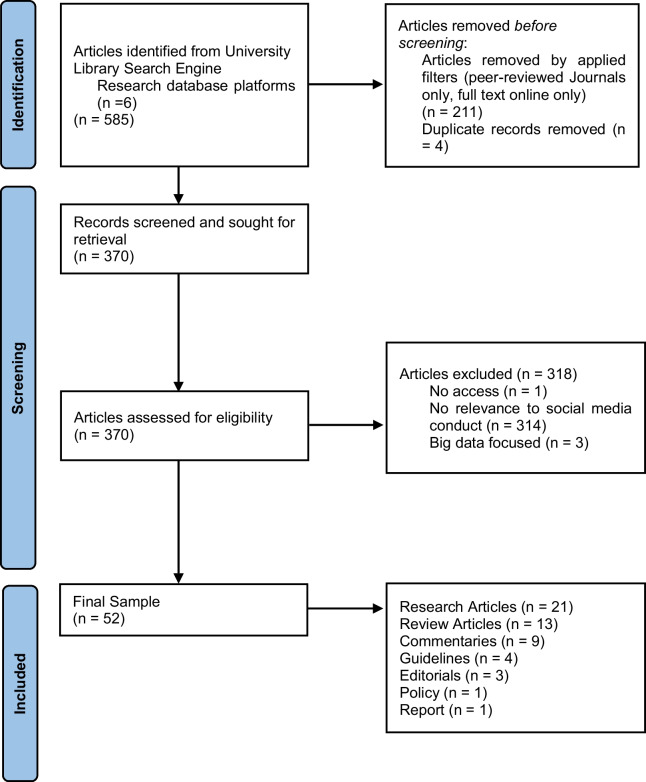
Summary of the identification of studies via research database platforms (PRISMA 2020 flow diagram)

The Preferred Reporting Items for Systematic reviews and Meta-Analyses (PRISMA) guidelines were used in this systematic literature review of relevant published articles (Fig. 1). PRISMA was chosen as it is a formal systematic review guideline, making it replicable and scientifically credible (Abelha et al. 2020; Shamseer et al. 2015). The PRISMA statement consists of a 27-item checklist and a flow diagram template to assist researchers in improving the reporting of systematic reviews (PRISMA 2021). PRISMA provides a comprehensive and consistent approach to systematically reviewing the literature, making it a popular choice among researchers.

In systematic reviews, data extraction is the process of creating a structured form based on key characteristics captured in the review process (Schmidt et al. 2021). Data extraction was carried out in this review by first developing an extraction form based on the research questions, objectives and inclusion criteria to record the author/year, what conduct problems can occur, what should a code of conduct include, what are the enablers of social media use, and article type. Pretesting of the data extraction process was conducted by the second author to assess the risk of bias. Then, articles available online were screened using the inclusion and exclusion criteria. Once the primary articles were identified, the relevant articles were extracted by the first author using the extraction form. The process involved reading the full text of each study. Once the data was extracted, the first author analysed and synthesised the data to answer the research questions. The end result of the extraction process is shown in Table 1.

**Table 1:** A summary of the final sample of articles

Author Year	What conduct problems can occur?	What should a code of conduct include?	What are the enablers of good social media use?	Article type
Jannsen 2009	Online profile can affect job prospects	Privacy		Guideline
Chilvers 2011	Abusive or inappropriate content	Privacy		Guideline
Ly and Ratnaike 2011	Breaching confidentiality		Process and procedure	Editorial
Childs and Martin 2012	Employee dilemmas	Separate personal from private. Privacy		Research article
Hughes 2012	Misconduct and unfit to practice			Policy
Knudson 2012	Privacy violations			Commentary
Sweet 2012			Reduce barriers to the use of social media	Commentary
Anderson and Guyton 2013	Employees sharing too much personal information			Review article
Holdsworth et al. 2013	Inappropriate employee conduct. Organisations seeing social media as disruptive		Staff need to use tool within an ethical framework and adhere to their own employee code of conduct	Research article
Suby 2013	Inappropriate use of photos and video			Commentary
Adams et al. 2014	Live tweeting during a medical procedure			Research article
Basevi et al. 2014	Privacy breaches. Blurring of personal and employee. Reputation damage	Separate personal and employee content online		Review article
Chinn 2014	People think private settings are private		Treating social media as a public space	Commentary
Fang et al. 2014	Confidentiality	Separating personal and employee		Research article
Jackson et al. 2014	Privacy	Employee filters, standards, privacy settings, setting boundaries		Guideline
Maloney et al. 2014		Peer collaboration, separate employee and personal, complimentary learning and enhanced communication		Research article
Moses et al. 2014	Blurring the boundaries between personal and employee social spheres	Self-auditing online	Trust	Review article
Smith and Lambert 2014	Blurring the lines between personal and employee use			Review article
Yap and Tiang 2014	Inability to separate personal from private use	Online professionalism		Review article
Gagnon and Sabus 2015	No one really steps out of their employee identity in their personal life	Separate personal and employee.		Commentary
Hunt et al. 2015		Employee conduct outweighs the right to contribute to social media freely		Review article
Osis and Pelling 2015	Blurring of boundaries between personal and employee		Training	Research article
Palacios-Gonzalez 2015	Privacy			Review article
Peate 2015		Privacy is key		Guideline
Peek et al. 2015			Develop employee and ethical guidelines	Review article
Alber et al. 2016			Training and education programs on how to use social media	Research article
Dhai and Grobler 2016	Blurring of boundaries between personal and employee. Privacy	Review privacy settings regularly. Review yourself regularly - how do you portray?		Commentary
Hamilton et al. 2016			Guidance, strategy, ongoing monitoring, education, training and support	Research article
Panahi et al. 2016	Privacy		Regulation and understanding by healthcare organisations	Research article
Patdu 2016		Separating personal and employee		Commentary
Peluchette et al. 2016	Separation of boundaries between personal and employee. Privacy		Need for a code of conduct for social networking sites	Research article
Swartz 2016	Permanent content			Editorial
Call and Hillock 2017	Personal vs professional life-balance	Separate personal from professional		Research article
Kubheka 2017		The right to freedom of speech must be limited to avoid violation of other people’s rights		Review article
Surani et al. 2017	Privacy			Research article
Holden and Spallek 2018	False information			Review article
Petersen and Lehmann 2018	Privacy			Editorial
Corniati et al. 2019			Educational and empowering paths towards good practices in social media	Research article
Popper-Giveon et al. 2019	Privacy. Reputation. Productivity		Formulate guidelines and develop a well-defined code of conduct	Research article
Terrasse et al. 2019	Searching for patient data online			Report
Ahmed et al. 2020	Privacy breach	Adapt behaviour to the digital world	Training	Research article
Al-Balushi 2020	Shared posts can be permanent. Inappropriate communication with patients	Separate personal and employee		Commentary
El Daouk et al. 2020			Organisational policies, best practices on managing social media	Research article
Ennis-O-Connor and Mannion 2020	Privacy	Actively monitor your online identity	Identify the goals of the organisation in the use of social media	Commentary
Ghalavand et al. 2020			Create and use suitable codes of conduct so healthcare organisation employees can use social media safely	Review article
Siegmund 2020	Privacy. False information. Hacking	Privacy settings. Awareness that anything post on social media can be discovered by employer		Review article
Bautista et al. 2021			Training	Research article
Comber et al. 2021	Privacy. Legal action	Separating personal from employee presence. Guidelines must allow healthcare organisation employees to use judgement but provide targeted and detailed direction	Training. Strict regulation may prevent health communication, so need a balance	Review article
Dailah and Naeem 2021			Social Media Organisational Productivity model	Research article
Khan et al. 2021	Privacy		Develop social media guidelines and strategies to improve interaction between healthcare organisations and consumers	Research article
Law et al. 2021		Confidentiality. Maintaining boundaries. Respect for colleagues and profession, raising concern, anonymity, conflict of interest	COVID-19 has provided healthcare organisations with an opportunity to update, clarify and align the strategic direction of using social media	Research article
Walsh et al. 2021	Privacy		Consider the risk and benefits	Research article

A keyword search was conducted using the authors’ university library search engine for journal articles in English published in the past 13 years from the following platforms: EBSCOhostEJS, ProQuest Central, Gale Academic OneFile, Science Citation Index Expanded (Web of Science), DOAJ Directory of Open Access Journals, and PubMed Central. The terms (‘social media’ OR ‘Facebook’ OR ‘social networking’) AND (‘health’ OR ‘healthcare’) were used to search article and journal titles, and the terms (‘employee’ OR ‘codes of’ OR ‘code of’) AND ‘conduct’ were used to search anywhere in the article, title, or journal title. While ‘social media’ is an important keyword in this review, alternative keywords such as ‘Facebook’ and ‘social networking’ have been included as they are popular alternatives to ‘social media’. Social media can also be referred to as ‘social networking sites’. It is important to recognise that not all organisations refer to their conduct guidelines the same way, and the terms ‘employee’, ‘code of’, and ‘codes of’ are frequently used by organisations.

The search identified 585 articles. A filter to retrieve only online full-text and peer-reviewed journal articles was applied, which removed 211 articles. Four duplicates were removed. The filtering process and removal of duplicates yielded a result of 370 articles. The title and abstract of each article were reviewed by author one and articles were excluded for the following reasons: no access, keyword only appeared in the references, or the article did not contain enough relevant literature to address the topic, specifically, the value of code of conduct in the use of social media in the health landscape. The use of the word ‘conduct’ resulted in articles using the term ‘conducted’ appearing in the search results. The word ‘conducted’ referred to a description of the research method. Some articles required a quick search of the keyword ‘conduct’ to confirm if the correct term for the systematic review was being used.

The screening process resulted in the removal of 318 articles, and the final shortlist included 52 articles. The final shortlist was checked by the second author to rule out bias and ensure that appropriate screening was conducted. Figure 1 provides a summary of the identification and screening process of the literature. The search results yielded a good mix of article types from 2009 to 2021 inclusively, and various article types to enhance the perspective of this literature review. A summary of the final sample of articles is provided in Table 1.

## Findings and discussion

In reviewing the literature, it is interesting to observe the evolution of concerns over the past 13 years with respect to how healthcare organisation employees conduct themselves on social media. There were 10 articles between 2009 and 2013 (the early years), and there was a noticeable increase in the literature from 2014 to 2015, at a time when social media in healthcare was dramatically growing (Fang et al. 2014). The years 2020 to 2021 showcased another noticeable increase in research, possibly affected due to the COVID-19 pandemic that forced healthcare organisations to increase their social media presence rapidly, and researchers wanting to report outcomes.

### Healthcare organisation employees using social media – what could go wrong?

The shortlisted literature was examined for the problems that can occur when healthcare organisation employees post health-related information either personally or professionally. Social media is ubiquitous in our society and has changed communication trends, offering a new channel to disperse information quickly and effectively (Chugh and Ruhi 2019) . Moreover, health communication via social media accelerated during the COVID-19 pandemic (Comber et al. 2021). Therefore, there is a need for healthcare organisation employees to understand how to use social media to avoid making mistakes that could damage reputations and result in termination of employment. Social media makes it possible to distribute information quickly to a broad audience and create a permanent electronic record that cannot be entirely deleted (Suby 2013; Swartz 2016). With the introduction of new technology and the unpredictability of human behaviour, there will be challenges, and new knowledge will be gained from those challenges, driving conduct on social media to adapt.

**Fig. 2:**
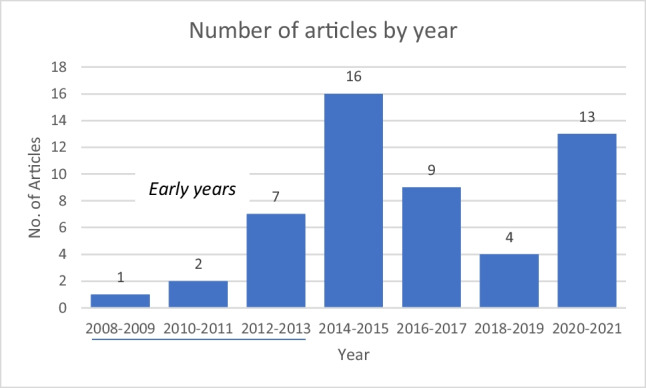
Number of articles in each 2-year period

Ten articles represented the early years of the use of social media in health (Fig. 2). The key problems found were privacy breaches (Anderson and Guyton 2013; Childs and Martin 2012; Chilvers 2011; Hughes 2012; Jannsen 2009; Ly and Ratnaike 2011; Suby 2013; Sweet 2012), the unclear distinction between personal and employee profiles (Childs and Martin 2012; Hughes 2012; Jannsen 2009; Knudson 2012; Ly and Ratnaike 2011; Smith and Lambert 2014) and abusive or inappropriate content (Chilvers 2011; Holdsworth et al. 2013; Hughes 2012; Suby 2013). Protecting patient privacy is paramount to the healthcare industry, and this resonated throughout the early years’ articles and continues to be a current problem (Comber et al. 2021; Khan et al. 2021; Law et al. 2021; Walsh et al. 2021). The issue of privacy focuses largely on breaching patient privacy, for which the healthcare industry has a strong revere. Concerns for employee privacy were raised as a risk that healthcare organisation employees share too much of themselves personally (Anderson and Guyton 2013) as they are not aware of the reach and permanency of social media posts.

Early years articles highlight the challenge of finding a balance between personal and professional use of social media (Childs and Martin 2012; Hughes 2012; Jannsen 2009; Ly and Ratnaike 2011) and has continued to be a problem as the right to personal freedom is not absolute (Call and Hillock 2017). The problem with the unclear distinction between personal and professional conduct is that whatever healthcare organisation employees post on social media is trusted (Law et al. 2021). The personal right to voice opinions and the permanency of social media posts does not bode well for healthcare organisation employees. Furthermore, employees who distribute information such as photos and videos of identifiable patients are liable in a court of law (Suby 2013). Personal information and personal views can damage reputations and have lasting consequences (Jannsen 2009). How healthcare organisation employees conduct themselves on social media can leave lasting impressions and also affect future job opportunities.

From 2014, more research articles emerged as healthcare organisations grappled with the use of social media but privacy and separating the boundaries between personal and professional conduct remained a continuing problem (Peluchette et al. 2016). A study to identify the ethical issues of live-tweeting during open-heart surgery revealed that the uses of social media in healthcare have not been fully examined, and healthcare organisations are unable to oversee and anticipate potential implications and need to weigh up the various aspects of use (Adams et al. 2014). New technology brings the opportunity to try new approaches, and only through testing boundaries will society discover success or failure, but without proper guidance, failures can be harmful.

Lack of knowledge about privacy settings was raised as a problem, suggesting that healthcare organisation employees cannot make informed decisions about posting content (Palacios-González 2015). In one study, participants were unaware of their appearance on Facebook and did not utilise sufficient privacy settings (Osis and Pelling 2015), making personal and professional conduct indistinguishable. However, privacy settings may enable a false understanding that posting privately remains private, as privacy settings guarantee the protection of personal data only to a certain extent (Adams et al. 2014; Chinn 2014). As a result, healthcare organisation employees may have a false sense of privacy that closed groups will keep their inappropriate behaviour hidden. However, a screenshot can be taken and distributed beyond a private group.

The right to personal freedom and the belief that personal social media posts remain personal is how healthcare organisation employees may make bad decisions when using social media and breach codes of conduct. Healthcare organisation employees have the right to have personal views but can never really step out of their professional identity in their personal life (Gagnon and Sabus 2015). Therefore, privacy continues to be a primary concern for healthcare organisations’ employee conduct (Ahmed et al. 2020; Comber et al. 2021; Khan et al. 2021; Petersen and Lehmann 2018; Siegmund 2020; Walsh et al. 2021), and caution must be exercised when employees post opinions or commentary in digital format in a public or private forum.

Other potential problems raised in the early years were inaccurate information becoming ‘fact’, loss of public trust and reputation management (Suby 2013). For example, in 2018, a study on the use of social media in the Australian dental profession found that compliance with National Law was poor and uncovered that false, misleading and deceptive information was posted on social media (Holden and Spallek 2018), demonstrating a lack of regulatory oversight. Furthermore, another problem identified was that healthcare organisation employees search for collateral information about their patients online (Terrasse et al. 2019). This issue was surprising as it contravenes patient privacy, and online information is not always accurate. Healthcare organisations should carefully consider the risk of employees using social media to search for patient data. This supports the notion that codes of conduct are critical to the successful education of staff and use of social media in healthcare.

### Elements of social media codes of conduct for healthcare organisations

The early years presented articles that attempted to provide guidelines to healthcare organisation employees on using social media to promote themselves on social media (Jannsen 2009) when social media was a new channel for communication in healthcare. However, in 2013, reports emerged from healthcare organisations of employees misusing social media, and the need for ethical frameworks became more apparent (Holdsworth et al. 2013). Moreover, codes of conduct needed to change.

The previous section demonstrates that maintaining privacy is the most important element for an effective social media code of conduct. A lack of awareness and knowledge of privacy settings (Osis and Pelling 2015; Palacios-González 2015) suggests that codes of conduct need to provide guidelines on understanding and using privacy settings (Basevi et al. 2014). Furthermore, privacy is the catalyst for separating personal and professional profiles.

A significant finding in the literature suggests that healthcare organisation employees should have separate personal and professional profiles (Al-Balushi 2020; Call and Hillock 2017; Comber et al. 2021; Fang et al. 2014; Gagnon and Sabus 2015; Jackson et al. 2014; Maloney et al. 2014; Moses et al. 2014; Osis and Pelling 2015; Patdu 2016) to avoid privacy breaches. Perhaps this thinking over the past decade has resulted in healthcare organisations still facing discipline issues with employees who display unacceptable behaviour on social media. Furthermore, separate accounts do not guarantee absolute privacy (Ennis-O-Connor and Mannion 2020). Maintaining separate profiles is a sensible approach to the issue of privacy. However, healthcare organisation employees need to understand that comments posted in a private forum can never be truly private. The decision to post comments that may breach codes of conduct needs to be considered.

It is important for healthcare organisation employees to understand that what can be said on social media is not always what you would say if you were face-to-face which is why codes of conduct specific to social media are required. Healthcare organisation employees need to consider with each post that they act with the same professionalism online as they would offline (Hughes 2012; Hunt et al. 2015). The social media guidance of the British Medical Association asserts that the professional obligation of healthcare organisation employees takes precedence over their freedom to participate in social media conversations (Hunt et al. 2015). The right to freedom of speech must be limited to avoid violating other people’s rights (Kubheka 2017). This notion goes to the heart of healthcare employees’ roles that the job must come first, which needs to be clear in codes of conduct. Furthermore, healthcare organisation employees should be aware that inappropriate posts on social media could be discovered by their employer (Siegmund 2020) and could result in disciplinary action or termination.

Fitness to practice and employability was raised (Yap and Tiang 2014) in the context of educating healthcare students on what to consider when posting online before being employed in healthcare organisations, such as posting content that would later embarrass them. In addition, healthcare organisation employees need to be careful about having a double standard for a professional image and personal image online and understand that it is not always easy to distinguish between personal and professional (Yap and Tiang 2014). This supports the notion that a good code of conduct will include raising awareness about how healthcare organisation employees are perceived online. Furthermore, a more progressive approach for healthcare organisations to reduce code breaches on social media is to include self-regulation and self-auditing in social media guidelines (Basevi et al. 2014; Call and Hillock 2017; Dhai and Grobler 2016; Ennis-O-Connor and Mannion 2020; Gagnon and Sabus 2015; Moses et al. 2014), encouraging healthcare organisation employees to regularly search themselves on the Internet and assess their own online presence. With a code of conduct to guide employees’, more self-awareness will be built, and more knowledge about how they are portrayed online will be gained, resulting in fewer breaches. Building self-awareness and knowledge promotes positive behaviours and reduces the risk of damaging reputation and loss of public trust.

Two articles (Basevi et al. 2014; Ennis-O-Connor and Mannion 2020) that contained specific guidelines for the use of social media in health were analysed. While the articles were published six years apart, it is interesting to note the similarities in Table 2. In summary, the key elements for an effective social media code of conduct in healthcare are:

1. Privacy
2. Exercise caution
3. Respect the profession
4. Self-review your profile and presence regularly
5. Commitment to following policy and guidelines
6. Commitment to continuous training and education

**Table 2:**
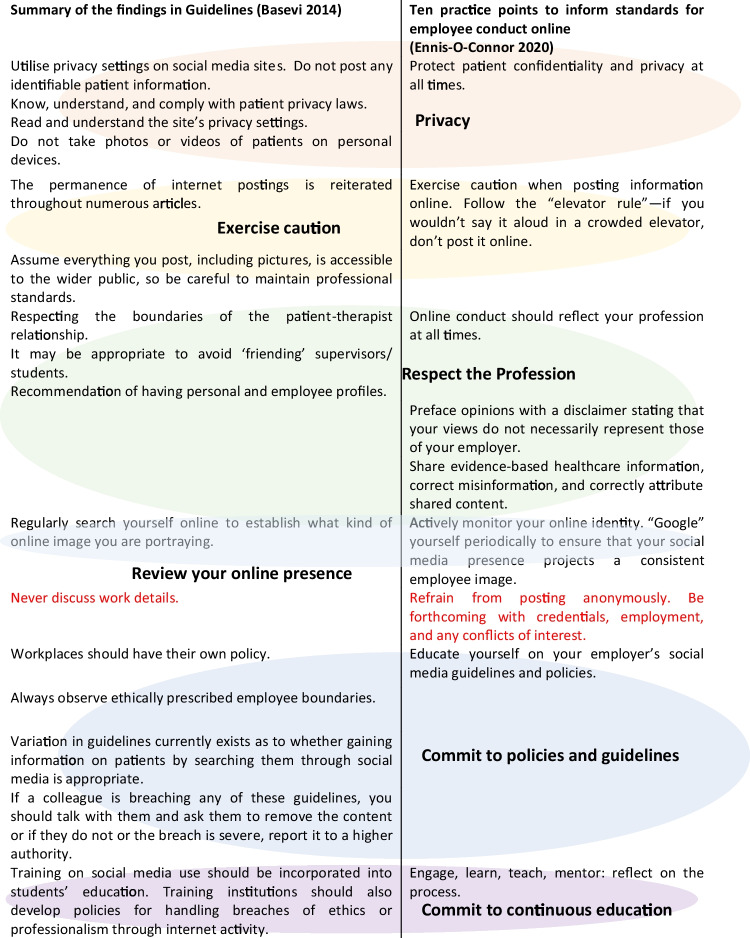
Comparison of two guidelines for elements that should be included in an effective social media code of conduct for healthcare organisation employees

The one difference between the two articles was never discussing work details (Basevi et al. 2014), which is the opposite of being forthcoming about your employment details (Ennis-O-Connor and Mannion 2020). It was unclear if the discussion of work details referred to personal posts, but research suggests that healthcare organisation employees should not post anonymously (Comber et al. 2021; Gagnon and Sabus 2015; Law et al. 2021).

### Enablers of good codes of conduct

Healthcare organisations have previously seen social media as disruptive and time-consuming (Suby 2013). The lack of established employee codes of conduct for social media (Hunt et al. 2015) could be explained because healthcare organisations did not consider that social media would become ubiquitous in society. Moreover, the effort to address social media codes of conduct would be wasted if social media in healthcare was not going to become pervasive. The lack of organisational support stemmed from privacy concerns, and the approach in the early years was to avoid social media use (Bautista et al. 2021). Avoiding social media created more issues for healthcare organisations as employees navigated ethical decisions themselves that resulted in breaches of the standard code of conduct (Clark and Kearns 2010).

Furthermore, a code of conduct that presents unreasonable barriers to the use of social media will likely result in more breaches. The COVID-19 pandemic has provided healthcare organisations with an opportunity to update, clarify and align the strategic direction of using social media (Law et al. 2021). Goals for the use of social media should be regularly reviewed (Ennis-O-Connor and Mannion 2020). It is essential that social media codes of conduct support the organisation’s values and goals and promote acceptable behaviours on social media.

Establishing codes of conduct for the use of new technology can be problematic if risks are not clearly identified, and problems can occur with how codes of conduct are written. In 2012, the Australian Health Practitioner Regulation Agency released a preliminary consultation paper on social media policy, suggesting that advertising regulations prohibit the use of testimonials in advertising, a conservative approach that had the potential to overlook the benefits (Sweet 2012).

#### Establish and review guidelines and codes of conduct

The findings in this review support the need for codes of conduct, policies and guidelines to be established specifically for the use of social media by healthcare organisation employees (Corniati et al. 2019; El Daouk et al. 2020; Ghalavand et al. 2020; Hamilton et al. 2016; Holdsworth et al. 2013; Khan et al. 2021; Peek et al. 2015; Peluchette et al. 2016; Popper-Giveon et al. 2019; Sweet 2012). With social media use in general, society is encouraged to take risks, be impulsive and be social. In healthcare taking risks can have harmful consequences. The nature of healthcare is to be conservative and protect the public. Furthermore, codes of conduct exist to protect healthcare organisation employees from harming others, themselves or the organisation’s reputation. Frameworks such as the Social Media Organizational Productivity Model (Dailah and Naeem 2021) will further assist healthcare organisations in utilising social media effectively and realising its benefits.

#### Training and education

Ongoing training and education are vital enablers of good social media conduct. Healthcare organisation employees are not always aware of current social media policies and codes (Collings-Hughes et al. 2021; Comber et al. 2021; Surani et al. 2017), thus increasing the risk of breaches. The findings in this review support the need for ongoing training and education in the use of social media for healthcare organisation employees (Ahmed et al. 2020; Alber et al. 2016; Bautista et al. 2021; Comber et al. 2021; Corniati et al. 2019; Hamilton et al. 2016; Osis and Pelling 2015). Healthcare organisation employees must adapt their behaviour to maintain professionalism in the digital age (Ahmed et al. 2020). Training and education programs need to include increasing awareness and understanding of codes and policies, guidelines, technology changes, and how to use the technology safely (such as using privacy settings). General communication training programs for healthcare organisation employees should also reflect how to effectively use social media for employee health communication (Bautista et al. 2021). Furthermore, healthcare organisation employees are not directly using social media in their work, and communication training programs will benefit all employees as the lines between personal and professional conduct can be unclear.

It can be argued that behaviour on social media is an extension of the standard code of conduct behaviour, respect and privacy. However, social media allows the lines between personal and employee online presence to be blurred easily. The call for healthcare organisations to establish social media codes of conduct started a decade ago (Anderson and Guyton 2013; Sweet 2012), focusing on using social media safely (Ghalavand et al. 2020). Healthcare organisations must develop, enforce and update policies to address appropriate and inappropriate conduct on social media, including employee agreements, orientation training, employee handbooks and performance appraisals (Suby 2013). Furthermore, ongoing monitoring of education, training and support needs to be addressed (Ahmed et al. 2020; Hamilton et al. 2016). Acknowledging that social media will continue to evolve and change will help healthcare organisations realise that an enabler of good social media codes of conduct is to continually educate, review and adapt.

#### Build a culture of awareness, respect and knowledge sharing and organisational support

Establishing a good healthcare organisation culture for the use of social media (Dailah and Naeem 2021) and a culture of knowledge sharing (Ghalavand et al. 2020) will enable social media codes of conduct to be used effectively. Healthcare organisations already undertaking social media-based consumer engagement activities should consider sharing methods and knowledge with other healthcare organisations (Walsh et al. 2021). It is vital that healthcare organisations acknowledge the risks and benefits of social media, address risks in codes of conduct and promote the benefits in a positive culture. Lack of organisational support is a barrier to using social media (Bautista et al. 2021). Organisational policies and structures often reflect the environmental expectations required to enhance social media use and increase success (Dailah and Naeem 2021). Furthermore, if the risks are not acknowledged, and appropriate usage strategies are not developed in codes of conduct, the risk of harm to patients, employees and the organisation will increase.

## Conclusion

Social media use in healthcare differs from other industries because healthcare information can be sensitive, often addressing public health issues such as disease or death. It is difficult for posts to be humorous or fun, and posts can be harmful if ambiguous, open to interpretation or false. Codes of conduct specific to social media use are to protect healthcare organisation employees from themselves and provide clearly articulated policies and rules around acceptable and unacceptable behaviours. It is essential that healthcare organisations identify breaches and address them accordingly and consistently. Employees might not like the rules, but if they are easy to follow and applied fairly, they will adhere to the codes of conduct.

This review examined the challenges of using social media for healthcare organisation employees, how codes of conduct can be used effectively and how conduct on social media may change for healthcare organisation employees in the future.

The key finding in this review focuses on privacy, particularly how healthcare organisation employees conduct themselves on social media. Furthermore, what constitutes a private social media post is not well understood by healthcare organisation employees, and while using common sense and personal judgement, the belief that social media posts are always private is false. To address privacy concerns, a second key finding in the literature was that healthcare organisation employees should maintain separate personal and professional accounts; however, this does not address the overall attitude toward how employees conduct themselves on social media. The third key finding was the need for codes of conduct and ongoing training and education to support those codes. Therefore, to enable the effective use of social media, it is recommended that all healthcare organisations establish social media-specific codes of conduct and implement a regular review cycle in conjunction with regular training and enforcement of values.

As with any review, this one also has limitations. For example, the search was limited to six research database platforms and used information from online full-text and peer-reviewed journal articles only. Hence, some relevant articles may have been omitted. In addition, while healthcare organisation websites may have provided codes of conduct, this paper aimed to review the existing literature that contributes to the field of enquiry. Furthermore, due to the changing healthcare landscape, it is also possible that new information on this topic may have been published since this paper was submitted for publication. Future research can fill these gaps.

## Data Availability

Data can be obtained from the corresponding author upon reasonable request.
